# Comparison of the Performance of Nonlinear Time-Dependent Constitutive Models Calibrated with Minimal Test Data Applied to an Epoxy Resin

**DOI:** 10.3390/ma18020404

**Published:** 2025-01-16

**Authors:** Rui Miranda Guedes, José Lopes Morais

**Affiliations:** 1 LAETA—Associate Laboratory of Energy, Transports and Aerospace, Department of Mechanical Engineering, Faculty of Engineering, University of Porto, 4200-465 Porto, Portugal; 2 LABIOMEP—Porto Biomechanics Laboratory, University of Porto, 4200-465 Porto, Portugal; 3 CITAB—Centre for the Research and Technology of Agro-Environmental and Biological Sciences, School of Science and Technology, University of Trás-os-Montes and Alto Douro, 5000-801 Vila Real, Portugal; jmorais@utad.pt

**Keywords:** epoxy resin, rheology properties, viscoelasticity, viscoplasticity, stress relaxation, multi-step, creep, constant strain rate

## Abstract

Epoxy resins are extensively employed as adhesives and matrices in fibre-reinforced composites. As polymers, they possess a viscoelastic nature and are prone to creep and stress relaxation even at room temperature. This phenomenon is also responsible for time-dependent failure or creep fracture due to cumulative strain. Several constitutive equations have been used to describe the mechanical time-dependent response of polymers. These models have been proposed over the past six decades, with minimal direct and practical confrontation. Each model is associated with a specific application or research group. This work assesses the predictive performance of four distinct time-dependent constitutive models based on experimental data. The models were deemed sufficiently straightforward to be readily integrated into practical engineering analyses. A range of loading cases, encompassing constant strain rate, creep, and relaxation tests, were conducted on a commercial epoxy resin. Model parameter calibration was conducted with a minimum data set. The extrapolative predictive capacity of the models was evaluated for creep loading by extending the tests to five decades. The selected rheological models comprise two viscoelastic models based on Volterra-type integrals, as originally proposed by Schapery and Rabotnov; one viscoplastic model, as originally proposed by Norton and Bailey; and the Burger model, in which two springs and two dashpots are combined in a serial and parallel configuration. The number of model parameters does not correlate positively to superior performance, even if it is high. Overall, the models exhibited satisfactory predictive performance, displaying similar outcomes with some relevant differences during the unloading phases.

## 1. Introduction

Epoxy resins have a wide range of applications, from the electronics industry, where they are used as encapsulants, to the building, automotive and aerospace industries, where they are used as structural adhesives and as matrices in fibre-reinforced composites. Its success is attributable to its excellent mechanical properties, high adhesion, heat resistance and electromagnetic insulation. The type of epoxy resin, hardener, and manufacturing process determine the resulting properties [1]. The long-chain molecular structure that is characteristic of polymers is the determining factor in their time-dependent response, a phenomenon referred to as “fading” memory [2]. Therefore, understanding and quantifying this phenomenon is relevant whenever dimensional stability is a concern or creep strain needs to be kept below critical levels under continuous load to avoid creep failure [3,4]. Time-dependent experimental characterisation is ordinarily an expensive and time-consuming process. A novel method using machine learning (ML) techniques has been developed to estimate the long-term creep modulus of thermoplastics using the open online database CAMPUS^®^. This method avoids the need for lengthy creep tests, thereby reducing the cost of obtaining the long-term creep modulus, an important design parameter for selecting polymers for long-term use [5]. However, ML techniques for predicting the long-term thermo-mechanical properties of materials are still in their infancy.

Due to the inherent properties of polymers, the use of viscoelastic or viscoplastic models is essential when analysing their time-dependent mechanical response. Therefore, it is important to have a comprehensive understanding of their constitutive behaviour under a range of loading conditions. Only then can an adequate prediction of their mechanical response under more general loading conditions be achieved.

In general, time-dependent materials are linear viscoelastic for sufficiently small strains, with the upper limit of linearity typically around 0.5% for many stiff polymers [6]. Above this threshold, the material starts displaying nonlinear behaviour, i.e., the response depends on the stress or strain state.

Different constitutive equations describe polymers’ mechanical response’s stress–strain time-dependency. These models have been put forth over the past six decades, with only a limited number of experimental and direct comparative assessments. Each model is associated with a specific application or research group. The constitutive models developed are generally expressed through integral or differential formulations. Classic examples of integral formulations for viscoelastic materials are the free-volume theory of Knauss and Emri [7] and the thermodynamic-based model of Schapery [8]. Differential formulation is the alternative type of constitutive equation easily adapted to finite element analysis [9,10]. Despite six decades of extensive research, there is no time-dependent constitutive model with global acceptance capable of predicting the mechanical response a polymer under complex loading histories [11,12,13,14]. Truly, the models denominated as unified viscoplastic theories were proposed for more complex loading conditions than creep or stress relaxation [15,16,17]. A double overstress (VBO) element configuration was developed for simulating strain rate sensitivity, creep, relaxation, and recovery behaviour, and addressing the non-monotonic changes in creep and relaxation that occur when a loading history involves some degree of unloading [14]. These advanced viscoplastic models involve large numbers of material parameters and naturally fit the experimental data better than the material models available in commercial finite element (FE) codes [16]. Since these approaches are not readily available in commercial FE code, it has inhibited their practical adoption [17].

Among several possibilities, four models were selected for this study: the Schapery single integral nonlinear constitutive model (SM) [8,11], the Burger’s model (BM) [18], the Norton–Bailey viscoplastic model (NB-SH) [19,20], and the nonlinear approach proposed by Rabotnov (Rabotnov) [21,22].

The integral formulation developed by Schapery [8], based on the irreversible thermodynamic approach for nonlinear viscoelastic materials, is one of the most quoted integral constitutive formulations [13]. The classical mechanical elements of mass, spring, and linear viscous dashpot have been employed as fundamental components in searching for more comprehensive mechanical models. The governing differential equations for these mechanical models provide differential constitutive equations that offer a phenomenological description of the viscoelastic response of polymers. The classical Burger’s model [18], i.e., the Maxwell and Kelvin elements connected in series, were used to describe the response of materials like asphalt [23] and soil beds [24]. The stress dependence of the model parameters is due to the nonlinear material response. Majda and Skrodzewicz [25] proposed Burger’s model, with the coefficients dependent on stress level, to represent the creep behaviour of an epoxy adhesive. However, the value of the modulus of elasticity (the spring of the Maxwell element) was assumed to be constant.

Another study [26] proposed modelling bonded joints using standard models supported by commercial Finite Element Method software (ANSYS version 12). In that context, the Bailey–Norton law [19,20] was selected to represent the creep response of an epoxy-based adhesive. A modified version of the Norton–Bailey model was proposed by Majda and Skrodzewicz [25]. The model parameters were assumed stress-dependent, to increase the degree of curve fitting of the experimental curves.

This work compares different nonlinear time-dependent constitutive models when applied to an epoxy resin. The selection criteria comprised two conditions. These models should be simple enough for industrial applications and present a universal character to capture the main features of the nonlinear viscoelasticity response. This experimental investigation was limited to the uniaxial stress state at room temperature.

The performance of the selected models was evaluated through a series of distinct loading conditions, encompassing constant strain rate, creep, and relaxation tests, applied to a commercial epoxy resin. Furthermore, the model parameters’ calibration was achieved with a minimum of experimental data. The number of material parameters differed between the models, with four for NB-SH and Rabotnov, seven for SM and eight for BM. Although the SM model achieved the best performance, the NB-SH model showed a good performance with few parameters.

## 2. Materials

The resin system used in this work was the SR 1500 epoxy, cured with SD 2503 hardener, and supplied by SICOMIN, Châteauneuf les Martigues, France. Accordingly, to the manufacturer, the formulation bases of SR 1500 epoxy are bisphenol A and F. This is an ambient temperature curing resin system, specifically developed for hand lay-up and vacuum bagging production. Plates with 200 × 200 × 2 mm were cast in a mould. The resin was cured by maintaining the polymer at 20 °C for 24 h, followed by 16 h at 60 °C. Later, the plates were subjected to a thermal post-cure, at 120 °C for 3 h, aiming at full curing. FTIR analysis confirmed no residual curing agent, or unreacted epoxy groups in the post-cured resin. The material was stored in a dry room for more than 12 months before being tested, long enough to reach the equilibrium at the glassy state. Briefly, cooling the epoxy resin from its rubbery state to a temperature lower than the glass transition temperature results in a non-equilibrium glassy state. This glassy state spontaneously evolves toward a temporally distant equilibrium, via slow molecular motions, under constant external conditions. Such a phenomenon is denominated as structural relaxation or physical ageing [27].

The test specimens with 80 × 6 × 2 mm^3^ were cut out from plates and the gauge length was 50 mm. The average tensile strength previously measured was 74 MPa. Three repetitions of the mechanical responses were obtained for each loading case performed at room temperature.

## 3. Experimental Tests

The test machine was an Instron^®^ ElectroPuls E1000 (High Wycombe, UK) with a 2 kN load cell. The data acquisition rate (displacement and load) was fixed at 2.5 Hz. Tests were performed at room temperature.

**Figure 1:**
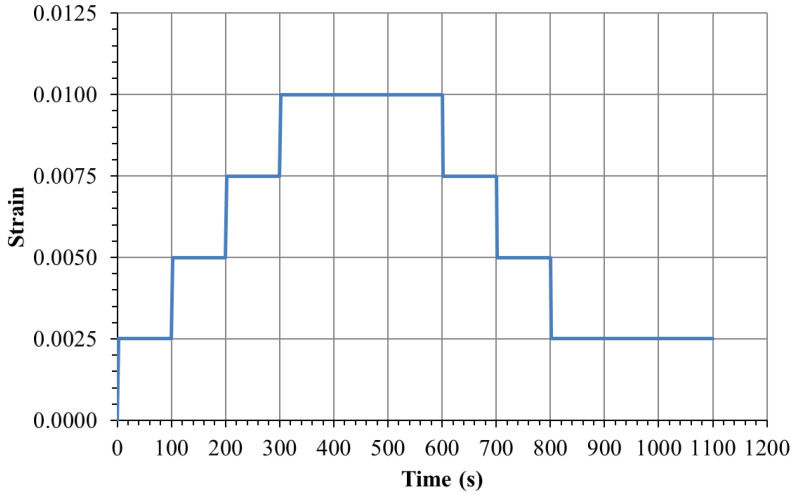
Step strain sequence (2 s ramp between each strain level) for the multi-step stress relaxation.

Eleven different types of tests were executed, as described in Table 1. These are grouped as, creep, constant strain rate (CSR) and stress relaxation. Three different multi-step loading cases were applied, as indicated in Table 2. The rise time varied from 0.4 to 1.0 s, imposed by a strain rate of 0.01/s. The last case was a multi-step relaxation with step strain evolution described in Figure 1.

## 4. Constitutive Time-Dependent Models

A description of the constitutive models is provided. Since we restricted this work to uniaxial loading cases, all the following descriptions are made accordingly. Nevertheless, the unidirectional model constitutive equations can be readily extended to the isotropic 3-dimensional formulation.

An important point must be made about the present approach. All the relationships presented are based on creep compliance, which predicts the strain response to variable uniaxial input stress. The alternative would have been to base the relationships on the relaxation modulus. Creep compliance and relaxation modulus are related for linear viscoelastic materials by an analytical expression. Schapery [8] claimed that there is no fundamental reason for a material to conform to both representations unless it is approximately elastic or linearly viscoelastic. However, the observed behaviour for a variety of materials is consistent with one or the other. Nevertheless, determining nonlinear parameters from relaxation tests is challenging and complex [28]. In response, several research papers have proposed methods to analytically or numerically determine the stress relaxation response directly from creep tests [28,29,30,31]. As Pupure et al. point out, the situation becomes more complicated when the viscoplastic strain becomes relevant [32].

In our case, stress relaxation tests are predicted from a limited amount of creep data. The experimental data used to calibrate the models was limited to creep at 6 and 24 MPa measured over 300 s. The CSR test at the highest strain rate was used to calibrate the nonlinear elastic parameters, assuming that the time-dependent effects are negligible at this rate. This test was not used for (NB-SH) as it does not include the nonlinear elastic behaviour. All other experimental data were used to evaluate the predictions of the models.

### 4.1. Schapery Model (SM) and Burger’s Model (BM)

These models have been described before but for completeness, a brief description of these two models is given in Appendix A. The SM [8] does not include damage and viscoplastic strain components. The latter are assumed to be non-recoverable, as discussed by Schapery in an updated analysis [33]. Nevertheless, the dashpot in the BM, which belongs to the Maxwell element, behaves as a viscoplastic component, as its strain is not recoverable unless an opposing stress is applied. To model the viscoelastic/viscoplastic behaviour of wood materials, the dashpot (of the Maxwell element in the Burger model) has been replaced by the Bingham element, which allows for a stress plasticity threshold [34]. The choice of components was made according to experimental observations. The aim was to reduce the number of parameters to a minimum.

### 4.2. Norton–Bailey Viscoplastic Model (NB-SH)

Bailey and Norton [19,20] developed a comprehensive model to describe the primary and secondary creep of metals at elevated temperatures. Singh and Mitchell proposed an enhanced expression for modelling the creep rate of soils, which was subsequently known as the Singh–Mitchell model [35]. Marry and Bray adopted this model to represent the time-dependent mechanical response of geomembranes made of high-density polyethylene, yielding favourable outcomes [36].

In this case, under a creep loading case, the total strain is given by

(1) $$\varepsilon =\frac{\sigma_{0}}{E}+Ae^{m\frac{\sigma \left(\tau \right)}{s_{0}}}\frac{1}{n+1}{\left(\frac{t}{\tau_{0}}\right)}^{n+1},$$

where *t* represents the time, *E* is the elastic modulus and *A*, *m*, *n* are the viscoplastic parameters. The parameters *s*_0_ and *t*_0_ are employed to assure a dimensionally coherent expression, in this case *s*_0_ = 1 MPa and *τ*_0_ = 1 h or *τ*_0_ = 1 s, i.e., depending on the unit of time used. Despite its empirical foundation, power law has been successfully employed in the analysis of polymers [37,38,39]. Fractional models provide a deeper meaning of the power law [40,41]. Modification of the Maxwell model, comprising a spring and a dashpot in series, by replacing the dashpot with a spring-pot (fractional model) leads to the power law for the creep loading condition [42].

The constitutive equations for viscoplastic strains under a general loading condition, depending on the strain state of the material at the time of the stress change, becomes

(2) $$\varepsilon_{vp}\left(t\right)=\frac{A}{n+1}{\left\{\frac{1}{\tau_{0}}\int_{0}^{t}e^{\frac{m\;\;\sigma \left(\tau \right)}{\left(n+1\right)\;\;s_{0}}}d\tau \right\}}^{n+1},$$

which is a strain-hardening formulation. Further details on these matters can be found in a previous work [43]. This model permits the incorporation of viscoplastic strain recovery during unloading stages. As observed by [44,45], the same viscoplastic model can represent the “positive” viscoplastic strains during loading and “negative” viscoplastic strains during unloading phases, noting that the direction of a viscous force is always opposite to the direction of displacement. While loading, a “positive” viscoplastic strain generates a viscous force acting in the opposite direction. Conversely, during unloading, the viscous force causes a “negative” viscoplastic strain. This effect explains the viscoplastic recovery, which may be partial.

### 4.3. Rabotnov Viscoelastic Model (Rabotnov)

Rabotnov [21] proposed an approach to construct a nonlinear equation as duly explained and tested using specimens made of polyoxymethylene [22]. Rabotnov assumed that all the nonlinearity may be gathered on the left-hand side of the equation

(3) $$\varphi \left(\varepsilon \right)=\sigma \left(t\right)+\int_{0}^{t}\frac{K}{n}{\left(\frac{t-\tau }{\tau_{0}}\right)}^{n}\frac{d\sigma \left(\tau \right)}{d\tau }d\tau ,$$

or after integrating by parts

(4) $$\varphi \left(\varepsilon \right)=\sigma \left(t\right)+\int_{0}^{t}K{\left(\frac{t-\tau }{\tau_{0}}\right)}^{n-1}\sigma \left(\tau \right)d\tau ,$$

where *τ*_0_ = 1 h or *τ*_0_ = 1 s, i.e., depending on the unit of time used, *K* and *n* are material parameters and *φ*(*ε*) represent the instantaneous or elastic stress–strain curve, in the limiting case when the stress (strain) rate tends to be infinite. The function was constructed using the CSR test data measured at the highest strain rate, with the assumption of two versions according to the case of imposed stress state or strain state, i.e.,

(5) $$\varepsilon =A\sigma^{2}+B\sigma ,$$

(6) $$\sigma =\varphi \left(\varepsilon \right)=a\varepsilon^{2}+b\varepsilon ,$$

where *A*, *B*, *a*, *b* are elastic constants of the material.

The conventional numerical methodology for Volterra-type integrals entails a conversion of the transient compliance to Prony’s series [46,47],

(7) $$K{\left(\frac{t}{\tau_{0}}\right)}^{n-1}=\sum_{i=1}^{N}k_{i}e^{-\frac{t}{\tau_{i}}},$$

consequently, at the time *t*, Equation (4) can be written as

(8) $$\varphi \left(\varepsilon \right)=\sigma \left(t\right)+\sum_{i=1}^{N}\int_{0}^{t}k_{i}e^{-\frac{t-\tau }{\tau_{i}}}\sigma \left(\tau \right)d\tau \;,$$

or

(9) $$\varphi \left(\varepsilon \right)=\sigma \left(t\right)+\sum_{i=1}^{N}F_{i}\left(\sigma ,t\right),$$

the subsequent time step calculation results in a recursive formulation,

(10) $$\varphi \left(\varepsilon \right)=\sigma \left(t+∆t\right)+\sum_{i=1}^{N}F_{i}\left(\sigma ,t+∆t\right),$$

where

(11) $$\begin{array}{ll} \sum_{i=1}^{N}F_{i}\left(\sigma ,t+∆t\right) & \;\\ \; & =\sum_{i=1}^{N}e^{-\frac{∆t}{\tau_{i}}}F_{i}\left(\sigma ,t\right)\\ \; & +\left(\frac{\sigma \left(t+∆t\right)+\sigma \left(t\right)}{2}\right)\sum_{i=1}^{N}k_{i}\tau_{i}\left(1-e^{-\frac{∆t}{\tau_{i}}}\right), \end{array}$$

in a more concise format

(12) $$\sum_{i=1}^{N}F_{i}\left(\sigma ,t+∆t\right)=Y\left(\sigma ,t\right)+\left(\frac{\sigma \left(t+∆t\right)+\sigma \left(t\right)}{2}\right)Z,$$

if the stress evolution is imposed, then the strain becomes

(13) $$\begin{array}{c} \varepsilon \left(t+∆t\right)=A{\left(\sigma \left(t+∆t\right)+Y\left(\sigma ,t\right)+\left(\frac{\sigma \left(t+∆t\right)+\sigma \left(t\right)}{2}\right)Z\right)}^{2}\\ \;\;\;\;\;\;+B\left(\sigma \left(t+∆t\right)+Y\left(\sigma ,t\right)+\left(\frac{\sigma \left(t+∆t\right)+\sigma \left(t\right)}{2}\right)Z\right), \end{array}$$

alternatively, if the strain evolution is imposed, then the stress becomes,

(14) $$\sigma \left(t+∆t\right)=\frac{\varphi \left(\varepsilon \left(t+∆t\right)\right)-2Y\left(\sigma ,t\right)-Z\sigma \left(t\right)}{2+Z}.$$

## 5. Experimental Results

The eleven distinct tests outlined in Table 1 and Table 2 and Figure 1, encompassing creep loading, constant strain rate (CSR), and stress relaxation, were successfully completed. The results for each load case are presented below. They are based on the average of three specimens.

The presentation of experimental results together with theoretical predictions is intended to avoid the repetition of plots. The following section is devoted to a comprehensive presentation of the methodology used to determine the model parameters.

### 5.1. Creep Tests

**Figure 2:**
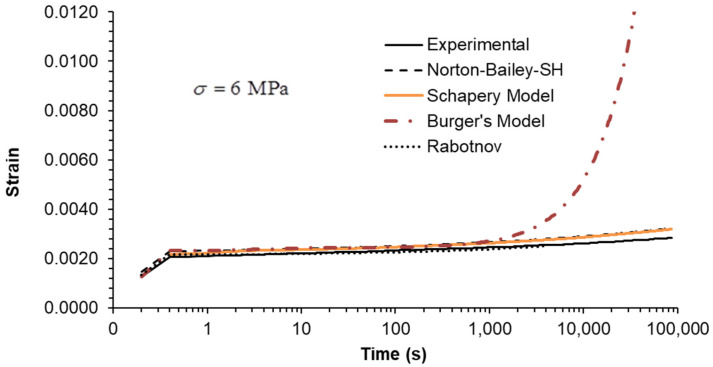
Creep test at 6 MPa during 24 h: experimental data compared against theoretical models (extrapolation from 300 s to 86,400 s).

**Figure 3:**
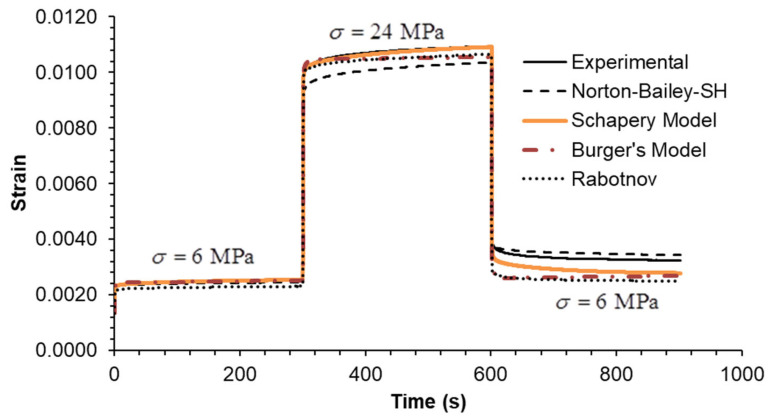
Case A: experimental data compared against theoretical models.

**Figure 4:**
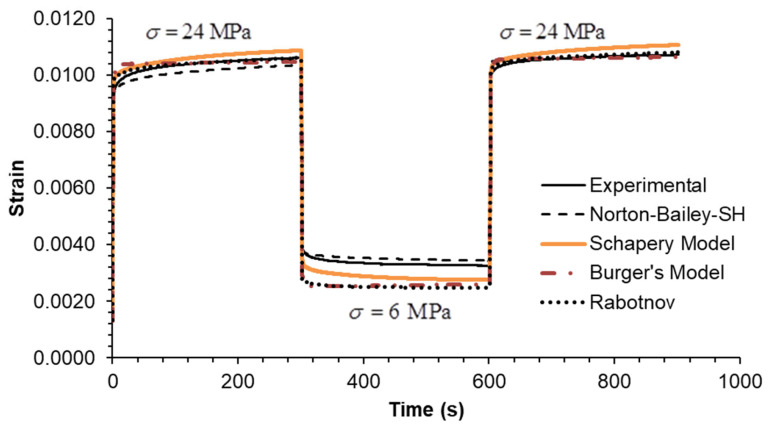
Case B: experimental data compared against theoretical models.

**Figure 5:**
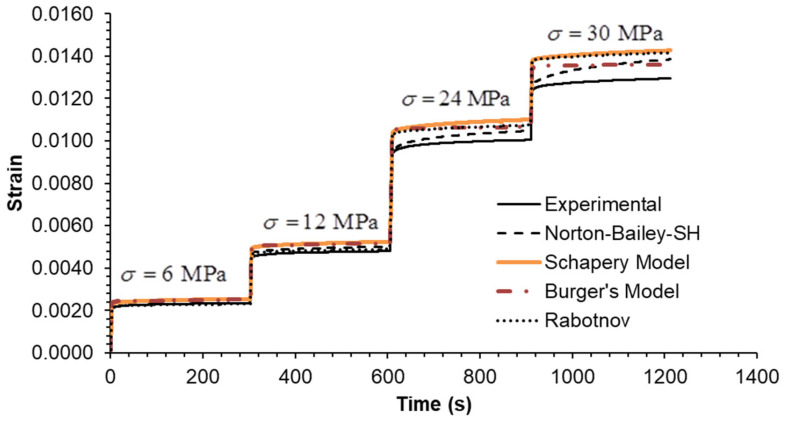
Case C: experimental data compared against theoretical models.

The predictive capability of the models was evaluated by extrapolation at creep loading by extending the tests to five decades. The creep test at 6 MPa was run for 86,400 s (equivalent to 24 h) as shown in Figure 2. It is evident that the BM is unable to accurately predict creep strain over timescales exceeding 1000 s. The long-term creep strain rate is influenced by the dashpot associated with the Maxwell element, which assumes a Newtonian viscosity law. The NB-SH and SM curves are in complete overlap throughout the entire plot, becoming almost indistinguishable from one another. This is near the experimental data, but not as close as the Rabotnov model. These models incorporate the creep power law function, which ensure reliable long-term creep extrapolations as observed in many cases [39]. An overview of the multi-step creep loading case results, plotted in Figure 3, Figure 4 and Figure 5, follows Table 3 containing the mean squared relative error ($MSRE=\frac{1}{n}\sum_{i=1}^{n}{\left(\frac{y_{i}^{exp}-y_{i}^{pred}}{y_{i}^{exp}}\right)}^{2}$) for each load step.

In Case A, all models demonstrate an MSRE value below 5.5% for each step. The largest discrepancies are observed in the final step during the recovery phase, except for the NB-SH model, which accounts for the recovery of the viscoplastic strain. In Case B, all models exhibit an MSRE value lower than 6.2%. The largest discrepancies are observed in the intermediate step 2, except for the NB-SH model, due to the same reason as previously stated. In Case C, all models exhibited MSRE values below 1%, thereby demonstrating comparable performance. The multi-step loading cases A and B showed greater discrepancies from the experimental data than case C. Consequently, the model predictions for the three multi-step loading cases were in reasonable agreement with the experimental data, except for the unloading phases. The exception was the NB-SH model.

Table 4 contains the MSRE values for the complete loading sequences. The respective Coefficients of Correlation (R^2^) between the observed and predicted data are given in Table 5.

### 5.2. Constant Strain Rate Tests

**Figure 6:**
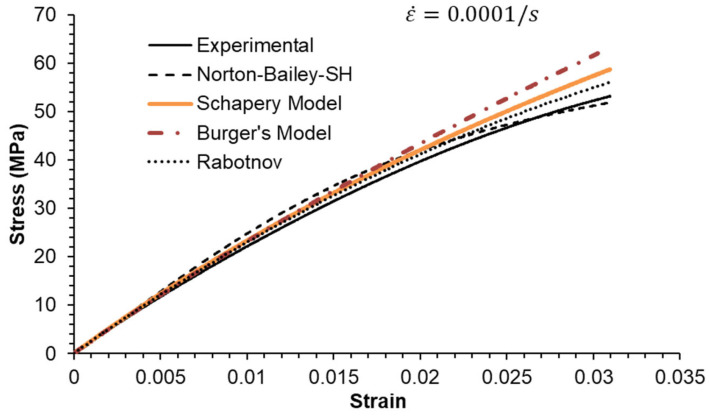
Constant strain rate of 0.0001/s: experimental data compared against theoretical models.

**Figure 7:**
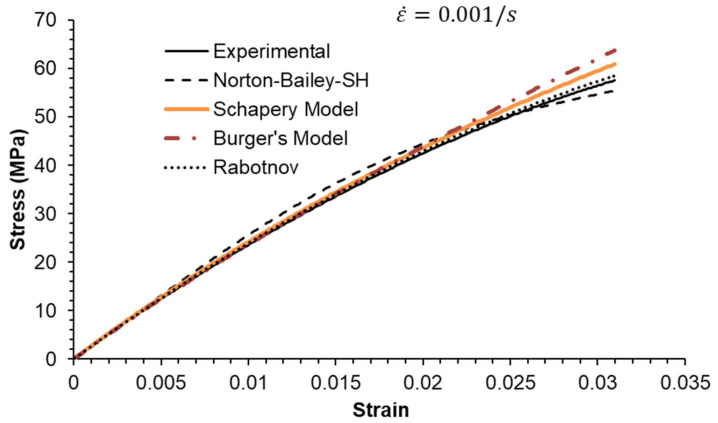
Constant strain rate of 0.001/s: experimental data compared against theoretical models.

**Figure 8:**
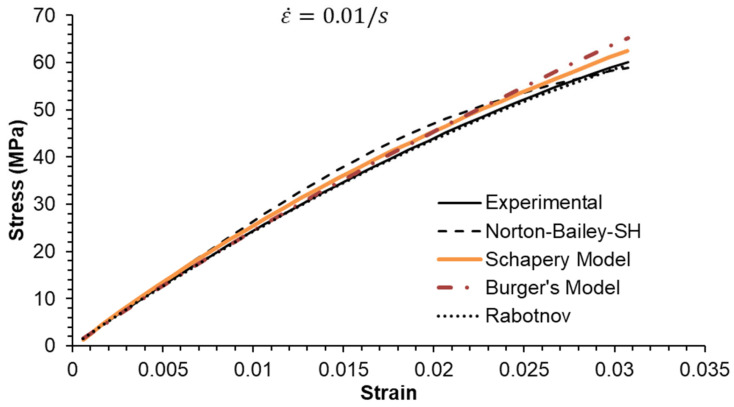
Constant strain rate of 0.01/s: experimental data compared against theoretical models.

Three strain rates separated by a factor of ten were used, as shown in Figure 6, Figure 7 and Figure 8. All MSRE values are below 4%, as documented in Table 4. The NB-SH shows the poorest performance as it is the only model calibrated using only the creep tests at 6 and 24 MPa. All other models show comparable performance with a high degree of correlation with experimental data as observed in Table 5.

The epoxy resin exhibits a pronounced nonlinear elastic effect, observable above the 0.5% strain threshold. This effect is not included in the NB-SH model as currently formulated. However, it was satisfactorily reproduced as a time-dependent phenomenon.

### 5.3. Stress Relaxation Tests

**Figure 9:**
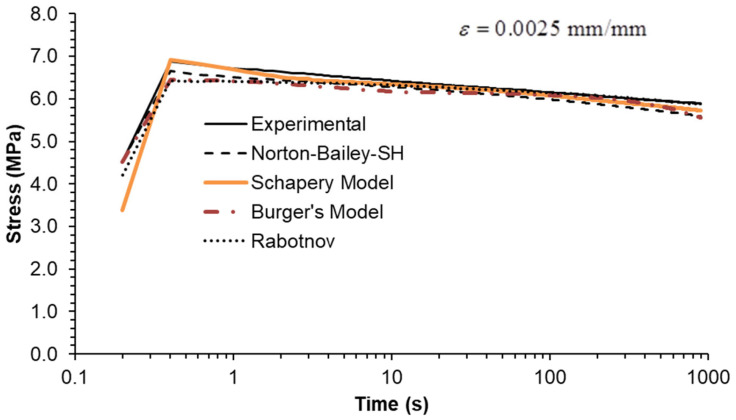
Stress relaxation under a constant strain of 0.0025: experimental data compared against theoretical models.

**Figure 10:**
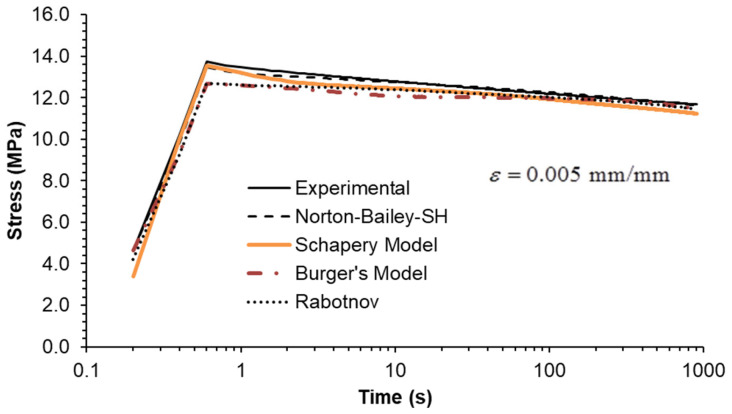
Stress relaxation under a constant strain of 0.005: experimental data compared against theoretical models.

**Figure 11:**
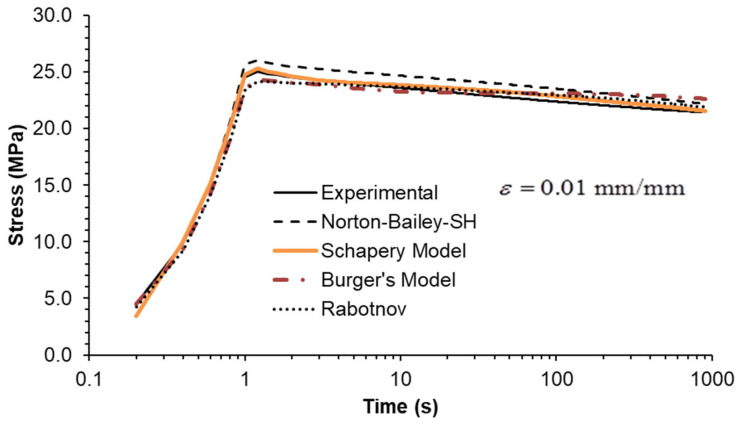
Stress relaxation under a constant strain of 0.01: experimental data compared against theoretical models.

Finally, stress relaxation tests were carried out at three different strain levels. As an ideal step strain cannot be applied by the testing machine, it was replaced by applying a constant strain rate ramp until the desired strain level was reached. Therefore, the rise time was included in the model calculations to closely follow the experiments. The inclusion of the rise time in the plots (Figure 9, Figure 10 and Figure 11) highlights this important detail.

The model predictions for each strain level are in good agreement with the experimental data, except for the NB-SH model at the higher strain level (Figure 9, Figure 10 and Figure 11). This is due to the pronounced nonlinear elastic effect not included in the model.

**Figure 12:**
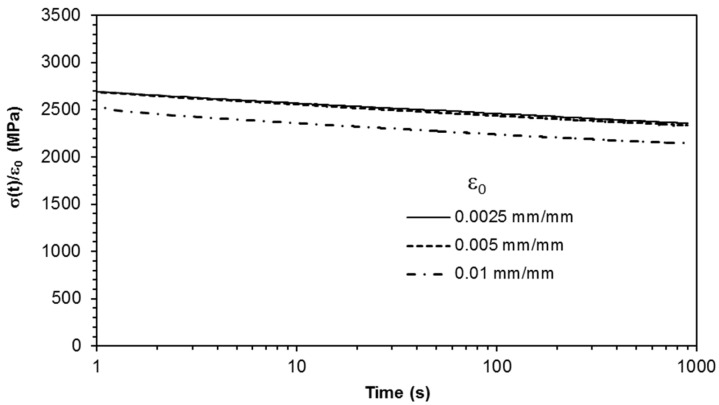
Comparison of normalised stress relaxation measured under different constant strain levels.

A linear behaviour below the 0.5% strain level is observed after plotting the normalised experimental stress relaxation data versus time (Figure 12). This is consistent with the observations from the CSR experimental data.

**Figure 13:**
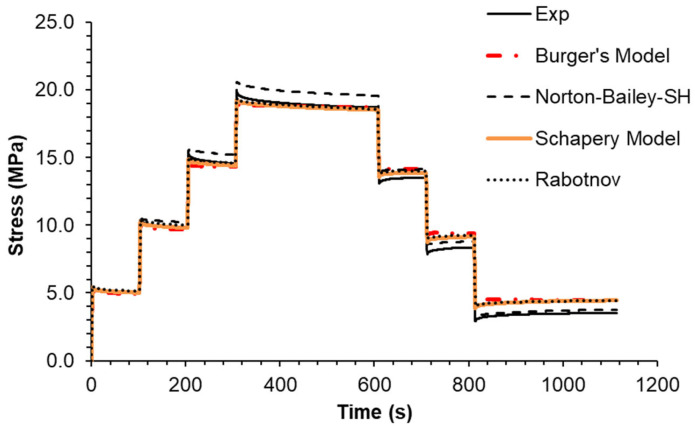
Multi-step stress relaxation test: experimental data compared against theoretical models.

The multi-stage relaxation test led to the conclusion that the performance of the NB-SH model was much better than the others (Figure 13). Although it predicts higher stress levels at the last two strain steps, the prediction at the unloading steps is remarkably close to the experimental data. The performance of the other models is quite similar with no significant differences.

## 6. Methodology Employed to Determine the Model Parameters

The adopted approach was based on creep strain data measured at 6 and 24 MPa during 300 s, to calibrate the model parameters through the curve fitting of analytical expressions. Since the parameters display stress dependency, linear and exponential stress functions were assumed for SM and BM, respectively. It was observed that the creep curves did not provide enough information about the nonlinear elastic behaviour. As the stress level reached around 60 MPa at constant strain rate, creep strain data measured at 6 and 24 MPa, regardless of time, cannot provide sufficient information about nonlinear elastic behaviour above 24 MPa. Accordingly, the constant strain rate curve (CSR) at the highest rate was employed to calibrate the nonlinear elastic parameters, except for the SB-SH model, which does not encompass the nonlinear elastic behaviour. The assumption was that the time-dependent effects would be negligible at the highest rate.

### Estimation of Parameters

The model parameters were determined following the methodology previously outlined. The SM parameters are presented in Table 6. The transient compliance (power law) was converted into a Prony series by imposing seven retardation times, 1/λ_i_, i = 1, 2, 3,…, 7 which accurately represents the 24 h period. The results are presented in Table 7. The BM parameters are illustrated in Table 8. In the present case, all the BM parameters are stress dependent. Appendix A contains a comprehensive explication of the parameters associated with the SM and BMs. The NB-SH parameters are shown in Table 9. As previously observed, the NB-SH includes the concept of a “positive” viscoplastic strain generated during the loading phase, which is responsible for the viscous force acting in the opposite direction, and a “negative” viscoplastic strain generated during the unloading phase, which opposes the unloading. The same model describes both events with the same parameters, except for one, as indicated. The Rabotnov model parameters are shown in Table 10. As was conducted previously for SM, the power law was converted into a Prony series, as shown in Table 11.

## 7. General Discussion

The maximum stress level attained at creep and stress relaxation tests was around 40% of the failure tensile stress. This stress level matches the in-service stress range applied to the material, assuming a safety factor of around 2. The nonlinear effects were observed above the 0.5% strain level, which is considered a typical value for polymers [6].

Earlier studies on epoxy resin-based composites at room temperature uncoupled the time-dependent deformation into viscoelastic and viscoplastic strains [28]. While the viscoelastic strain is recoverable during and after the unloading, the viscoplastic strain remains unrecoverable [29,31,36,47]. Although no specific tests were conducted to obtain the viscoplastic strains of this epoxy resin, from the previous studies it should be expected a viscoelastic–viscoplastic response. Still, the applied models being purely viscoelastic (SM, BM, and Rabotnov) or purely viscoplastic (NB-SH), were able to capture all the experimental data in a unified manner. This was more evident for the last model, since it distinguishes the loading and unloading states. This phenomenon has been the subject of detailed description and modelling by Ellyin et al. [12,48]. Another note concerns the BM where the mechanical response of the dashpot in series acts as a viscoplastic component, i.e., its cumulative strain is unrecoverable.

In the case of NB-SH, the model calibration was based on the creep tests only. This epoxy shows nonlinear elastic behaviour, but the NB-SH does not include this type of response.

The quality of model predictions, measured globally by the MSRE (Table 10), allows us to observe the overall differences in model performance. The corresponding Coefficients of Correlation (R^2^) confirm the overall good quality of the models’ predictions. In summary, SM performs better than the other models. The number of model parameters plays a role in the ability of the model to capture the experimental data. These considerations apply when the model is used to fit the experimental data. However, it is not certain that the model predictions would simply improve by increasing the number of parameters. In the present case, the number of parameters varied from four for NB-SH and Rabotnov to seven for SM and eight for BM. The NB-SH offers a very good compromise between the number of parameters and its performance. In addition, the NB-SH would benefit from the inclusion of the nonlinear elastic effect.

## 8. Conclusions

Four viscoelastic models were selected to assess its relative performance using experimental data obtained for an epoxy resin. These tests comprise a set of different loading cases, including constant strain rate, creep, and relaxation tests.

A methodology was established to perform the parameters calibration of the viscoelastic models, based on a limited amount of creep experimental data up to 300 s. The remaining data were used to assess the prediction ability of the models.

The observed trends were correctly predicted by all the models. Additionally, the creep tests were extended over five decades, and the models were able to predict correctly the creep strain after 24 h. The unique exception was BM; decidedly, it cannot be used to extrapolate long-term creep strain. Altogether, the four constitutive models describe in a unified manner the time-dependent mechanical response of epoxy resin under various loading modes, i.e., creep, constant strain rate, and stress relaxation.

The quality of predictions was evaluated through the mean squared relative error (MSRE) and Coefficients of Correlation (R^2^); SM distinctively performs better than the other models. Alternatively, NB-SH offers a good compromise between the number of parameters and its performance.

Prospects for investigating the performance of these models will include temperature effects and, at a later stage, extending the models to biaxial loading, since in many situations it can be assumed that the polymers are subjected to a plane stress state.

## Figures and Tables

**Table 1:** Index of all tests that have been carried out.

Test Type	Case Nr.	Test Description
Creep	1	Test at 6 MPa during 24 h
	2	Multi-step loading case A
	3	Multi-step loading case B
	4	Multi-step loading case C
CSR	5	Constant Strain Rate, 0.0001/s
	6	Constant Strain Rate, 0.001/s
	7	Constant Strain Rate, 0.01/s.
Stress Relax	8	Constant Strain, 0.0025 mm/mm
	9	Constant Strain, 0.005 mm/mm
	10	Constant Strain, 0.01 mm/mm
Multi-step Relaxation	11	Described in Figure 1

**Table 2:** Multi-step loading tests description.

Case A	Stress (MPa)	6	24	6	
	Dwell Time (s)	300	300	300	
Case B	Stress (MPa)	24	6	24	
	Dwell Time (s)	300	300	300	
Case C	Stress (MPa)	6	12	24	30
	Dwell Time (s)	300	300	300	300

**Table 3:** Mean squared relative error (MSRE) calculated for all models and multi-step loading cases.

Case	Step	BM	NB-SH	SM	Rabotnov
	1	0.00012	0.00072	0.00001	0.00684
A	2	0.00064	0.00336	0.00002	0.00056
	3	0.04205	0.00319	0.01642	0.05510
	1	0.00042	0.00065	0.00050	0.00009
B	2	0.05453	0.00261	0.02069	0.06175
	3	0.00042	0.00009	0.00082	0.00032
	1	0.00620	0.00924	0.00614	0.00023
C	2	0.00665	0.00178	0.00779	0.00047
	3	0.00548	0.00108	0.00863	0.00504
	4	0.00464	0.00199	0.00957	0.00715

**Table 4:** Mean squared relative error (MSRE) calculated for all models and all loading cases.

Test Type	Case Nr.	BM *	NB-SH	SM	Rabotnov
Creep	1	0.00615	0.00904	0.00661	0.00153
	2	0.01434	0.00242	0.00551	0.02088
	3	0.01838	0.00111	0.00731	0.02065
	4	0.00552	0.00393	0.00752	0.00378
CSR	5	0.00972	0.03956	0.00536	0.00319
	6	0.00189	0.00438	0.00113	0.00029
	7	0.00146	0.00378	0.00176	0.00017
Stress Relax	8	0.00083	0.00155	0.00044	0.00002
	9	0.00024	0.00001	0.00094	0.00022
	10	0.00255	0.00188	0.00021	0.00070
Multi-step Relaxation		0.02912	0.00251	0.02107	0.02199
MSRE Sum		0.06109	0.06766	0.03679	0.07342

* Predictions after 1000 s were not considered for Case 1.

**Table 5:** Coefficients of correlation (R^2^) between the observed and predicted data.

Test Type	Case Nr.	BM *	NB-SH	SM	Rabotnov
Creep	1	0.84169	0.99014	0.99131	0.92453
	2	0.99340	0.99906	0.99743	0.99561
	3	0.99817	0.99833	0.99692	0.99954
	4	0.99958	0.99902	0.99964	0.99969
CSR	5	0.99614	0.99259	0.99923	0.99991
	6	0.99749	0.99364	0.99965	0.99999
	7	0.99850	0.99380	0.99993	0.99998
Stress Relax	8	0.78597	0.97890	0.98601	0.95147
	9	0.82708	0.98150	0.98512	0.95191
	10	0.70199	0.98279	0.98146	0.95144
Multi-step Relaxation		0.99361	0.99977	0.99511	0.99789
Average R^2^		0.92124	0.99178	0.99380	0.97927

* Predictions after 1000 s were not considered for Case 1.

**Table 6:** Schapery model parameters (power law).

*D*_0_	*D*_1_	*n*
MPa^−1^		
3.543 × 10^−4^	2.570 × 10^−5^	0.17
g_0_ = 5.6433 × 10^−3^σ + 0.93703		
g_2_ = 0.0065884σ + 0.96047		

**Table 7:** Prony series calculated after the power law.

	1/s		MPa^−1^
-	-	D_0_	3.5430 × 10^−4^
l_1_	0.000001	D_1_	1.2838 × 10^−4^
l_2_	0.00001	D_2_	6.2690 × 10^−5^
l_3_	0.0001	D_3_	4.1898 × 10^−5^
l_4_	0.001	D_4_	2.8442 × 10^−5^
l_5_	0.01	D_5_	2.0994 × 10^−5^
l_6_	0.1	D_6_	8.5457 × 10^−6^
l_7_	1	D_7_	3.0854 × 10^−5^

**Table 8:** Burger’s model parameters.

R_1_ =	$2.6497\;\times \;10^{3}e^{{-3.0452\times 10}^{-3}\sigma }$	(MPa)
h_1_ =	$1.3702\;\times \;10^{7}e^{{7.7016\times 10}^{-2}\sigma }$	(MPa·s)
R_2_ =	$5.0442\;\times \;10^{4}e^{{-1.2602\times 10}^{-2}\sigma }$	(MPa)
h_2_ =	$2.0177\;\times \;10^{5}e^{{-1.2602\times 10}^{-2}\sigma }$	(MPa·s)

**Table 9:** Norton–Bailey (NB-SH) parameters.

*E*	*m*	*n*	*A* *
(MPa)	-	-	(MPa)^(1−m)^
2772	0.08324	−0.8300	1.5909 × 10^−5^

* During unloading, A′ = A/2.

**Table 10:** Rabotnov’s parameters (power law).

*A*	*B*	*a*	*b*	*K*	*n*
1/(MPa)^2^	1/(MPa)	(MPa)	(MPa)	-	-
−22,952	2662	2.739 × 10^−5^	3.386 × 10^−4^	0.007000	−0.8000

**Table 11:** Prony series for Rabotnov’s model.

	*s*		
t_1_	2	k_1_	6.7199 × 10^−3^
t_2_	20	k_2_	1.4547 × 10^−3^
t_3_	200	k_3_	1.3459 × 10^−4^
t_4_	2000	k_4_	5.5475 × 10^−5^

**Figure A1:**
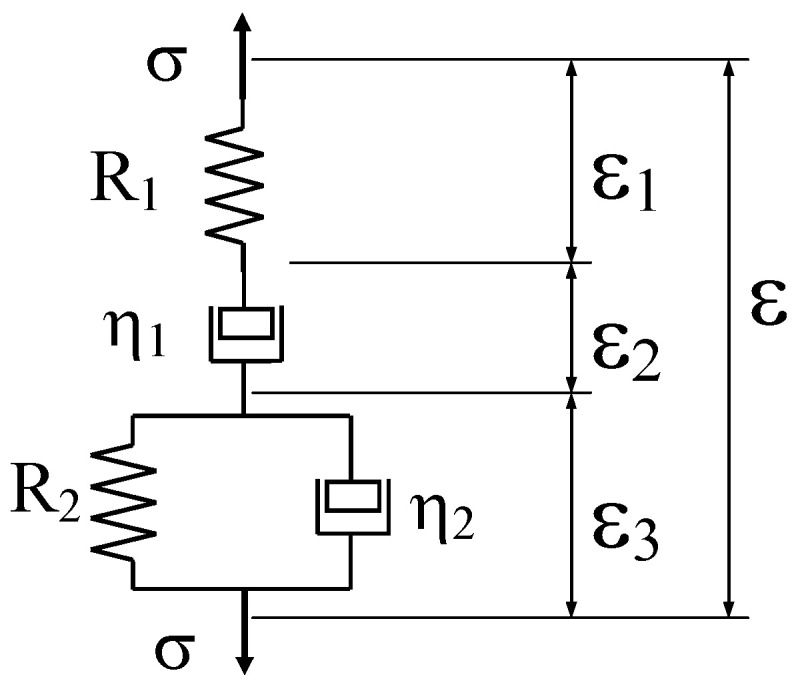
Burger’s model.

## Data Availability

The original contributions presented in this study are included in the article. Further inquiries can be directed to the corresponding author.

## Appendix A

### Appendix A.1. Schapery Model (SM)

The nonlinear viscoelastic strain–stress relationship for the uniaxial loading condition induced by an arbitrary stress history is given by [8]

(A1) $$\varepsilon \left(t\right)=D_{0}g_{0}\sigma \left(t\right)+g_{1}\int_{0}^{t}\Delta D\left(\psi -\psi^{’}\right)\frac{d\left(g_{2}\sigma \left(\tau \right)\right)}{d\tau }d\tau ,$$

where *D*_0_ is the elastic compliance and the kernel Δ*D*(*t*) is the time dependent compliance, the correspondent reduced times *Ψ* and *Ψ*′ given by

(A2) $$\Psi =\int_{0}^{t}\frac{d\tau^{’}}{a_{\sigma }}\;,\;\Psi^{’}=\int_{0}^{\tau }\frac{d\tau^{’}}{a_{\sigma }},$$

where *g*_0_, *g*_1_, *g*_2_, *a*_σ_ are four stress-dependent nonlinearising parameters.

In the present case, the material model used is a simplification of the Schapery equation by imposing *g*_1_ = *a*_σ_ = 1, and the kernel Δ*D*(*t*) is given by the power law

(A3) $$\varepsilon \left(t\right)=D_{0}g_{0}\sigma \left(t\right)+\int_{0}^{t}D_{1}{\left(\frac{t-\tau }{\tau_{0}}\right)}^{n}\frac{d\left(g_{2}\sigma \left(\tau \right)\right)}{d\tau }d\tau ,$$

where *D*_1_ and the exponent *n* are material parameters. The creep response of the model, i.e., the response to *σ* = *σ*_0_ for *t* ≥ 0, is

(A4) $$\varepsilon \left(t\right)=D_{0}g_{0}\sigma_{0}+D_{1}g_{2}{\left(\frac{t}{\tau_{0}}\right)}^{n}\sigma_{0}.$$

For general loading cases the strain history was calculated via Equation (A3), after eliminating the Volterra-type integrals, by converting the transient compliance to Prony’s series [46,47]

(A5) $$\varepsilon \Delta D\left(t\right)=\sum_{i=1}^{N}D_{i}\left(1-e^{-\lambda_{i}t}\right),$$

where *D_i_*, *λ_i_* are linear viscoelastic parameters. Substituting (A5) into (A3), we obtain

(A6) $$\varepsilon \left(t\right)=D_{0}g_{0}\sigma \left(t\right)+\sum_{i=1}^{N}\int_{0}^{t}D_{i}\left(1-e^{-\lambda_{i}\left(t-\tau \right)}\right)\frac{d\left(g_{2}\sigma \left(\tau \right)\right)}{d\tau }d\tau .$$

After algebraic manipulations, the following recursive formula is obtained [31]:

(A7) $$\varepsilon \left(t\right)=g_{0}D_{0}\sigma \left(t\right)+\sum_{i=1}^{N}\varepsilon_{i}\left(t\right),$$

where

$$\varepsilon_{i}\left(t_{j+1}\right)=e^{-\lambda_{i}\Delta t}\cdot \varepsilon_{i}\left(t_{j}\right)+\left[1-\frac{1}{\lambda_{i}\Delta t}\left(1-e^{-\lambda_{i}\Delta t}\right)\right]D_{i}\;g_{2}\sigma \left(t_{j+1}\right)\;+\left[\frac{1}{\lambda_{i}\Delta t}\left(1-e^{-\;\lambda_{i}\Delta t}\right)-e^{-\;\lambda_{i}\Delta t}\right]D_{i}\;g_{2}\sigma \left(t_{j}\right)$$

with

$$\Delta t=t_{j+1}-t_{j}.$$

The total strain given by Equation (A7), can then be written as

(A8) $$\varepsilon \left(t_{j+1}\right)=g_{0}D_{0}\sigma \left(t_{j+1}\right)+R\left(t_{j+1}\right),$$

where

(A9) $$R\left(t_{j+1}\right)=g_{1}\sum_{i=1}^{N}\varepsilon_{i}\left(t_{j+1}\right).$$

The previous formulation allows us to calculate the strain under any given stress history. If the strain history is given, then the stress history must be determined. Equation (A8) can be used to do the following:

(A10) $$\sigma \left(t_{j+1}\right)=\frac{\varepsilon \left(t_{j+1}\right)-R\left(t_{j+1}\right)}{g_{0}D_{0}}.$$

This formula becomes an implicit equation since $R\left(t_{j+1}\right)$ depends on the stress $\sigma \left(t_{j+1}\right)$ level.

### Appendix A.2. Burger’s Model (BM)

In the elementary mechanical models, a spring for elastic behaviour and a dashpot for viscous behaviour can be combined to obtain differential stress–strain relations. The four-element model, known as Burger’s model, has been used to describe the creep behaviour of epoxy resins [25]. Burger’s model is a combination of the Maxwell and Kelvin-Voigt models in series, as shown in Figure A1.

The total strain is given as

(A11) $$\varepsilon =\varepsilon_{1}+\varepsilon_{2}+\varepsilon_{3},$$

where

(A12) $$\varepsilon_{1}=\frac{\sigma }{R_{1}},{\dot{\varepsilon }}_{2}=\frac{\sigma }{\eta_{1}}\;and\;{\dot{\varepsilon }}_{3}+\frac{R_{2}}{\eta_{2}}\varepsilon_{3}=\frac{\sigma }{\eta_{2}}.$$

The dot stands for derivation to *t* (time). Eliminating the internal variables (*ε*_1_, *ε*_2_, *ε*_3_), the following differential equation is obtained:

(A13) $$\sigma +\left(\frac{\eta_{1}}{R_{1}}+\frac{\eta_{1}}{R_{2}}+\frac{\eta_{2}}{R_{2}}\right)\dot{\sigma }+\left(\frac{\eta_{1}\eta_{2}}{R_{1}R_{2}}\right)\ddot{\sigma }=\eta_{1}\dot{\varepsilon }+\frac{\eta_{1}\eta_{2}}{R_{2}}\varepsilon .$$

The response of the model to the case of creep, i.e., constant load condition *σ* = *σ*_0_ for *t* ≥ 0, is

(A14) $$\varepsilon =\frac{\sigma_{0}}{R_{1}}+\frac{\sigma_{0}}{\eta_{1}}t+\frac{\sigma_{0}}{R_{2}}\left(1-e^{-\frac{R_{2}}{\eta_{2}}t}\right).$$

Yet, the integration for a general loading history can be performed incrementally, using finite differences, through the internal variables (*ε*_1_, *ε*_2_, *ε*_3_)

(A15) $$\varepsilon \left(t_{j+1}\right)=\varepsilon_{1}\left(t_{j+1}\right)+\varepsilon_{2}\left(t_{j+1}\right)+\varepsilon_{3}\left(t_{j+1}\right).$$

The total strain can be obtained recursively by using finite differences

(A16) $$\begin{array}{c} \varepsilon_{1}\left(t_{j+1}\right)=\frac{1}{R_{1}}\sigma \left(t_{j+1}\right),\;\varepsilon_{2}\left(t_{j+1}\right)=\frac{\Delta t}{\eta_{1}}\sigma \left(t_{j+1}\right)+\varepsilon_{2}\left(t_{j}\right)\\ \;\;\mathrm{and}\;\varepsilon_{3}\left(t_{j+1}\right)=\frac{\left[\frac{\Delta t}{\eta_{2}}\sigma \left(t_{j+1}\right)+\varepsilon_{3}\left(t_{j}\right)\right]}{\left(1+\frac{R_{2}}{\eta_{2}}\Delta t\right)}, \end{array}$$

or

(A17) $$\begin{array}{c} \varepsilon \left(t_{j+1}\right)=\frac{1}{R_{1}}\sigma \left(t_{j+1}\right)+\frac{\Delta t}{\eta_{1}}\sigma \left(t_{j+1}\right)+\varepsilon_{2}\left(t_{j}\right)\\ \;\;\;\;+\frac{\left[\frac{\Delta t}{\eta_{2}}\sigma \left(t_{j+1}\right)+\varepsilon_{3}\left(t_{j}\right)\right]}{\left(1+\frac{R_{2}}{\eta_{2}}\Delta t\right)}. \end{array}$$

It is possible to invert the calculation, i.e., imposing $\varepsilon \left(t_{j+1}\right)$ and calculating $\sigma \left(t_{j+1}\right)$

(A18) $$\sigma \left(t_{j+1}\right)=\frac{\left[\varepsilon \left(t_{j+1}\right)-\varepsilon_{2}\left(t_{j}\right)-\varepsilon_{3}\left(t_{j}\right)\frac{\eta_{2}}{\eta_{2}+R_{2}\Delta t}\right]}{\left(\frac{1}{R_{1}}+\frac{\Delta t}{\eta_{1}}+\frac{\Delta t}{\eta_{2}+R_{2}\Delta t}\right)}.$$

Burger’s model predicts, on a long-term basis, an unlimited strain increases at a constant strain rate. However, the experimental findings for polymers observed continuous strain increase with decreasing strain rate, complying with the Findlay’s power law [37].

## References

[1] Jin F.L., Li X., Park S.J. (2015). Synthesis and application of epoxy resins: A review. J. Ind. Eng. Chem..

[2] Brinson H.F., Brinson L.C. (2008). Polymer Engineering Science and Viscoelasticity: An Introduction.

[3] Guedes R.M. (2004). Mathematical analysis of energies for viscoelastic materials and energy based failure criteria for creep loading. Mech. Time-Depend. Mater..

[4] Kontou E., Spathis G. (2014). Viscoplastic response and creep failure time prediction of polymers based on the transient network model. Mech. Time-Depend. Mater..

[5] Lobato H., Cernuda C., Zulueta K., Arriaga A., Matxain J.M., Burgoa A. (2024). Prediction of long-term creep modulus of thermoplastics using brief tests and interpretable machine learning. Int. J. Solids Struct..

[6] McCrum N., Buckley C.P., Bucknall C.B. (1997). Principles of Polymer Engineering.

[7] Knauss W.G., Emri I. (1987). Volume change and the nonlinearly thermo-viscoelastic constitution of polymers. Polym. Eng. Sci..

[8] Schapery R.A. (1969). On the characterization of nonlinear viscoelastic materials. Polym. Eng. Sci..

[9] Chen Y., Xia Z., Ellyin F. (2001). Evolution of residual stresses induced during curing processing using a viscoelastic micromechanical model. J. Compos. Mater..

[10] Yi S., Hilton H.H. (1997). Free edge stresses in elastic and viscoelastic composites under uniaxial extension, bending, and twisting loadings. J. Eng. Mater. Technol. Trans. ASME.

[11] Schapery R.A. (2000). Nonlinear viscoelastic solids. Int. J. Solids Struct..

[12] Ellyin F., Xia Z. (2006). Nonlinear viscoelastic constitutive model for thermoset polymers. J. Eng. Mater. Technol. Trans. ASME.

[13] Jamshidi M., Shokrieh M.M. (2024). On the Schapery nonlinear viscoelastic model: A review. Eur. J. Mech. A/Solids.

[14] Khan F., Yeakle C. (2011). Experimental investigation and modeling of non-monotonic creep behavior in polymers. Int. J. Plast..

[15] Chiu W.K., Jones R. (1995). Unified constitutive model for thermoset adhesive, FM73. Int. J. Adhes. Adhes..

[16] Yu X.X., Crocombe A.D., Richardson G. (2001). Material modelling for rate-dependent adhesives. Int. J. Adhes. Adhes..

[17] Scott M.L., Elder D.J., Feih S., Gunnion A.J., Liu X.L., Thomson R.S. (2010). Engineering solutions for complex composite material behaviour spanning time and temperature scales. Philos. Mag..

[18] Burgers J.M. (1939). Mechanical Considerations—Model Systems—Phenomenological Theories of Relaxation and of Viscosity.

[19] Norton F.H. (1929). The Creep of Steel at High Temperatures.

[20] Bailey R.W. (1935). The utilization of creep test data in engineering design. Proc. Inst. Mech. Eng..

[21] Rabotnov Y.N. (1980). Elements of Hereditary Solid Mechanics.

[22] Suvorova J.V., Ohlson N.G., Alexeeva S.I. (2003). An approach to the description of time-dependent materials. Mater. Des..

[23] Muliana A., Rajagopal K.R., Wineman A. (2013). On the response of Burgers’ fluid and its generalizations with pressure dependent moduli. Mech. Time-Depend. Mater..

[24] Dey A., Basudhar P.K. (2010). Applicability of Burger Model in Predicting the Response of Viscoelastic Soil Beds.

[25] Majda P., Skrodzewicz J. (2009). A modified creep model of epoxy adhesive at ambient temperature. Int. J. Adhes. Adhes..

[26] Zehsaz M., Vakili-Tahami F., Saeimi-Sadigh M.A. (2014). Creep analysis of adhesively bonded single lap joint using finite element method. J. Mech. Sci. Technol..

[27] Hutchinson J.M. (1995). Physical aging of polymers. Prog. Polym. Sci..

[28] Pupure L., Varna J., Joffe R. (2018). Methodology for macro-modeling of bio-based composites with inelastic constituents. Compos. Sci. Technol..

[29] Oza A., Vanderby R., Lakes R.S. (2003). Interrelation of creep and relaxation for nonlinearly viscoelastic materials: Application to ligament and metal. Rheol. Acta.

[30] Touati D., Cederbaum G. (1997). On the prediction of stress relaxation from known creep of nonlinear materials. J. Eng. Mater. Technol. Trans. ASME.

[31] Guedes R.M., Marques A.T., Cardon A. (1998). Analytical and Experimental Evaluation of Nonlinear Viscoelastic-Viscoplastic Composite Laminates under Creep, Creep-Recovery, Relaxation and Ramp Loading. Mech. Time-Depend. Mater..

[32] Pupure L., Pakrastins L., Varna J. (2021). Challenges in developing of 3D nonlinear viscoelastic models. IOP Conf. Ser. Mater. Sci. Eng..

[33] Schapery R.A. (1997). Nonlinear Viscoelastic and Viscoplastic Constitutive Equations Based on Thermodynamics. Mech. Time-Depend. Mater..

[34] Fothe T., Azeufack U.G., Kenmeugne B., Fogue M. (2022). A one-dimensional elasto-viscoplastic model coupled to damage for the description of creep in wooden materials. J. Wood Sci..

[35] Singh A., Mitchell J.K. (1968). General stress-strain-time function for soils. J. Soil Mech. Found. Div..

[36] Merry S.M., Bray J.D. (1997). Time-dependent mechanical response of HDPE geomembranes. J. Geotech. Eng..

[37] Findley W.N., Lai J.S., Onaran K. (1989). Creep and Relaxation of Nonlinear Viscoelastic Materials: With an Introduction to Linear Viscoelasticity.

[38] Findley W.N. (1987). 26-Year creep and recovery of poly(vinyl chloride) and polyethylene. Polym. Eng. Sci..

[39] Scott D.W., Lai J.S., Zureick A.H. (1995). Creep Behavior of Fiber-Reinforced Polymeric Composites: A Review of the Technical Literature. J. Reinf. Plast. Compos..

[40] Heymans N., Bauwens J.C. (1994). Fractal rheological models and fractional differential equations for viscoelastic behavior. Rheol. Acta.

[41] Glöckle W.G., Nonnenmacher T.F. (1991). Fractional Integral Operators and Fox Functions in the Theory of Viscoelasticity. Macromolecules.

[42] Guedes R.M. (2011). A viscoelastic model for a biomedical ultra-high molecular weight polyethylene using the time-temperature superposition principle. Polym. Test..

[43] Guedes R.M. (2023). A practical model to predict the time-dependent behaviour of angle-ply laminates from limited creep data. Mech. Time-Depend. Mater..

[44] Kim W., Sun C.T. (2002). Modeling relaxation of a polymeric composite during loading and unloading. J. Compos. Mater..

[45] Guedes R.M. (2009). Viscoplastic analysis of fiber reinforced polymer matrix composites under various loading conditions. Polym. Compos..

[46] Czyz J.A., Szyszkowski W. (1990). An effective method for non-linear viscoelastic structural analysis. Comput. Struct..

[47] Tuttle M.E., Pasricha A., Emery A.F. (1995). The Nonlinear Viscoelastic-Viscoplastic Behavior of IM7/5260 Composites Subjected to Cyclic Loading. J. Compos. Mater..

[48] Ellyin F., Vaziri R., Bigot L. (2007). Predictions of two nonlinear viscoelastic constitutive relations for polymers under multiaxial loadings. Polym. Eng. Sci..

